# Longitudinal Adipokine and Lipid Profiles in Fabry Disease

**DOI:** 10.3390/jcm15062390

**Published:** 2026-03-20

**Authors:** Constantin Gatterer, Daniela Allmer, Dietrich Beitzke, Senta Graf, Philipp Hohensinner, Markus Ponleitner, Eva Steinacher, Alice Schmidt, Gere Sunder-Plassmann, Paulus Rommer, Max Lenz

**Affiliations:** 1 Division of Cardiology, Department of Internal Medicine II, Medical University of Vienna, 1090 Vienna, Austria; constantin.gatterer@meduniwien.ac.at (C.G.); daniela.allmer@meduniwien.ac.at (D.A.); senta.graf@meduniwien.ac.at (S.G.); eva.steinacher@meduniwien.ac.at (E.S.); 2 Division of Nephrology and Dialysis, Department of Internal Medicine III, Medical University of Vienna, 1090 Vienna, Austria; gere.sunder-plassmann@meduniwien.ac.at; 3 Department of Biomedical Imaging and Image-Guided Therapy, Medical University of Vienna, 1090 Vienna, Austriaalice.schmidt@meduniwien.ac.at (A.S.);; 4 Center for Biomedical Research and Translational Surgery, Medical University of Vienna, 1090 Vienna, Austria; 5 Department of Neurology, Medical University of Vienna, 1090 Vienna, Austria

**Keywords:** Fabry disease, Fabry cardiomyopathy, adiponectin, leptin, NT-proBNP, enzyme replacement therapy, lipid profile, sex differences

## Abstract

**Background/Objectives:** Fabry disease (FD) is a lysosomal storage disorder characterized by progressive renal and cardiac involvement and an increased burden of cardiovascular and cerebrovascular events. While cardiac magnetic resonance imaging (CMR) has significantly advanced structural assessment, circulating biomarkers reflecting disease-related cardiac manifestations remain incompletely understood. We therefore investigated adiponectin and leptin, two adipokines involved in inflammatory, metabolic, and fibrotic pathways, in relation to cardiac involvement and analyzed long-term lipid trajectories in FD. **Methods:** This longitudinal observational study included 49 patients with FD with 149 study visits. Circulating adiponectin, leptin, NT-proBNP, and conventional lipid parameters were assessed longitudinally and stratified by FD-specific therapy status and sex. Multivariable linear regression was performed to evaluate independent associations with log-transformed NT-proBNP values. **Results:** Adiponectin was positively associated with NT-proBNP, reflecting cardiac involvement, independent of age, sex, BMI, and eGFR (*p* < 0.001). Higher adiponectin levels were observed in patients with left ventricular hypertrophy or low T1 and those with fibrosis, detected by CMR (*p* = 0.009 and *p* < 0.001, respectively). This association was mainly seen in patients receiving FD-specific therapy, raising the question of whether this reflects underlying organ involvement or treatment effects. Leptin demonstrated weaker, inverse associations. Adiponectin, leptin, Triglycerides, total cholesterol, and HDL- and LDL-cholesterol levels remained stable over long-term follow-up, irrespective of FD-specific therapy or sex. **Conclusions:** In FD, adiponectin appears to be associated with cardiac involvement, and conventional lipid parameters remained unchanged over time. These findings suggest that alterations in adipokines, rather than progressive dyslipidemia, may reflect disease-related cardiac manifestations.

## 1. Introduction

Fabry disease (FD; Online Mendelian Inheritance in Man [OMIM] #300644) is a rare X-linked lysosomal storage disorder caused by a deficiency of α-galactosidase A, due to variants in the *GLA* gene, resulting in progressive accumulation of glycosphingolipids within lysosome-containing cells [1]. The disease manifests as a multi-organ disorder affecting the kidneys, nervous system, and heart. Cardiac involvement occurs in up to 60% of affected males and approximately 50% of females during the disease course [2,3,4,5]. Fabry cardiomyopathy is characterized by concentric left ventricular hypertrophy, progressive myocardial remodeling, microvascular dysfunction, and, at advanced stages, replacement fibrosis [5,6,7]. Due to their widespread availability, echocardiography and the electrocardiogram (ECG) still play important roles in the diagnosis of cardiac involvement in FD [8,9]. More recently, however, Cardiac magnetic resonance imaging (CMR), including parametric mapping and late gadolinium enhancement (LGE), has substantially improved the non-invasive assessment of myocardial involvement. However, imaging and ECG alone may not fully capture dynamic aspects of disease progression, particularly in early or pre-hypertrophic stages [10,11]. Circulating biomarkers reflecting the underlying pathophysiology and treatment effects may therefore complement imaging-based disease staging and risk stratification. Beyond myocardial remodeling, FD is associated with endothelial dysfunction, chronic low-grade inflammation, oxidative stress, impaired sympathetic innervation, and an increased risk of cardiovascular and cerebrovascular events [5,6,7,12,13,14,15,16].

Beyond conventional lipid parameters, adipose tissue-derived hormones (adipokines) have emerged as relevant modulators of cardiovascular diseases and contribute to pathophysiological pathways similar to those in FD [17,18,19,20]. This suggests shared pathophysiological mechanisms with metabolically driven cardiovascular disease. Adiponectin exerts anti-inflammatory and anti-fibrotic effects, whereas leptin has been linked to hypertrophy, sympathetic nervous system activation, and adverse cardiovascular outcomes, particularly under metabolic and inflammatory stress [21]. Notably, an inverse association between adiponectin levels and heart failure, including those with preserved ejection fraction (HFpEF), has been described, also referred to as the “adiponectin paradox” [18]. However, the pattern of elevated adiponectin levels being linked to impaired functional capacity, diastolic dysfunction, microvascular dysfunction, and worse outcomes has not yet been fully explained [22]. Potentially underlying mechanisms include the development of adiponectin receptor insensitivity, disturbances in intracellular signaling pathways under persistent inflammatory conditions, and the possibility that elevated levels arise secondary to hemodynamic burden rather than serving as a potentially causal factor [18,23].

Given that Fabry cardiomyopathy is driven by the accumulation of glycosphingolipids rather than primary metabolic dysregulation, it remains unclear whether adipokine patterns observed in other cardiovascular diseases are applicable to this population. It is known that conventional risk factors such as hypercholesterolemia contribute significantly to cardiovascular event rates in FD [4]. However, data on adipokines in FD remain limited. A study by Hovakimyan et al. found no overall difference in plasma adiponectin between patients with or without FD [24]. However, they found an association between lower adiponectin values and cardiovascular involvement in male patients. Conversely, higher adiponectin levels were observed in male patients with renal involvement, and yet, long-term data on adipokines and their association with disease course in FD are currently lacking and are to be explored in the current study. Furthermore, our study aimed to examine whether lipid profiles differ by FD-specific therapy status, whether they change over time, and whether sex-specific differences exist in this X-linked disorder.

## 2. Materials and Methods

### 2.1. Patient Inclusion and Follow-Up

This study includes prospectively enrolled patients with *GLA* gene variants from the Fabry registry “KarMA” (cardiac manifestations of Fabry-Anderson disease—Kardiale Manifestation bei Morbus Anderson-Fabry) treated at the Medical University of Vienna. Patients were consecutively recruited between October 2011 and May 2024. All individuals provided written informed consent prior to inclusion. Only patients aged ≥18 years were eligible. Both individuals with and without treatment indication were included to reflect the heterogeneous clinical presentation and course of FD. Follow-up visits were scheduled according to disease severity and clinical necessity, typically on an annual basis. For longitudinal analyses, visits were clustered into predefined time windows of 30 (±15), 60 (±15), 90 (±15), and >105 months following baseline. If multiple or unscheduled visits occurred within a given time window, the visit closest to the respective time point was selected for analysis. Unless otherwise specified, all available follow-up data were included in the respective analyses. The study protocol was approved by the institutional ethics committee of the Medical University of Vienna (Nr: 1867/2021 and 813/2010) and was conducted in accordance with the Declaration of Helsinki and its subsequent amendments.

### 2.2. Clinical Assessment and Laboratory Measurements

At each visit, clinical characteristics, medical history, and therapy status were recorded. Moreover, Fabry-associated organ involvement was systematically assessed. Cardiac and renal manifestations were evaluated using established biomarkers and imaging modalities in accordance with institutional standards and current recommendations [25,26]. Routine laboratory parameters were analyzed at the central laboratory of the Vienna General Hospital using standardized protocols. For biobanking, peripheral venous blood samples were collected using two serum separator tubes, two EDTA tubes, and two 3.8% sodium citrate tubes (Greiner Bio-One, Kremsmünster, Austria). Samples were centrifuged at 3000 rpm at 4 °C, aliquoted, and consecutively stored at −80 °C until further analysis. EDTA blood samples were used for the preparation of dried blood spot (DBS) cards (ARCHIMED Life Science GmbH, Vienna, Austria, and Centogene GmbH, Rostock, Germany). The DBS cards were subsequently sent to the respective laboratories for analysis. Lyso-Gb3 concentrations were quantified using mass spectrometry as previously described [27]. Reference values were defined as <3.5 ng/mL for ARCHIMED Life Science and ≤1.8 ng/mL for Centogene analyses. Adiponectin and leptin concentrations were quantified using commercially available ELISA kits (Human Leptin DuoSet ELISA and Human Adiponectin/Acrp30 DuoSet ELISA, both R&D Systems, Minneapolis, MN, USA) according to the manufacturer’s instructions. The estimated glomerular filtration rate (eGFR) was calculated using the race-free CKD-EPI equation. [28]

### 2.3. Cardiac Imaging

Transthoracic echocardiography (TTE) and CMR were performed during scheduled follow-up visits. CMR examinations were performed at 1.5 Tesla using a Siemens Avanto Fit scanner (Siemens Healthineers AG, Erlangen, Germany) according to a standardized multiparametric protocol [29]. Post-processing was performed using dedicated software (Medis Suite MR 4.0.70.4, Medis Medical Imaging, Leiden, The Netherlands). Left and right ventricular volumes and function, as well as left atrial volume, were derived from cine images using standardized post-processing protocols as previously described [30]. Native T1 mapping was performed in the mid-ventricular septum. Values < 950 ms were defined as low T1 [31]. For LGE imaging, gadobutrol (Gadovist^®^, Bayer, Berlin, Germany) was administered at a dose of 0.15 mL/kg body weight. TTE (2D and Doppler) examinations were performed in the echocardiography laboratory at the Vienna General Hospital using commercially available ultrasound systems (Vivid E95, Vivid S70; General Electric Healthcare, Chicago, IL, USA) equipped with a 3.5 MHz transducer. All examinations adhered to the current recommendations of the American Society of Echocardiography and the European Association of Cardiovascular Imaging [32]. Left ventricular systolic function was visually estimated. Left ventricular hypertrophy (LVH) was defined as interventricular septal thickness ≥12 mm on TTE or as increased LV mass index (LVMI, excluding papillary muscles) >75 g/m^2^ in men and >59 g/m^2^ in women on CMR [33]. Cardiac involvement was classified as “none”, “intermediate” (T1 reduction and/or left ventricular hypertrophy without LGE), or “advanced” (presence of LGE in CMR).

### 2.4. Statistical Analysis

Categorical variables are reported as counts and percentages and were compared using the Chi-square test or Fisher’s exact test, as appropriate. Continuous variables are presented as median and interquartile ranges (IQR) or mean (±standard deviation). Normality was assessed visually as well as with the Kolmogorov–Smirnov-test, whilst homogeneity of variances was evaluated by Levene’s test. Accordingly, parametric data were compared using the unpaired Student’s *t*-test, whereas non-parametric variables were analyzed using the Mann–Whitney U test. Comparisons across multiple groups were performed using one-way ANOVA followed by Bonferroni-corrected post hoc tests. Correlations were assessed using Spearman’s rank correlation coefficient (r). Multivariable linear regression analysis was performed to assess the independent association between log-transformed NT-proBNP and adiponectin, adjusted for age, sex, BMI, and eGFR. All statistical analyses were performed using SPSS Statistics version 29.0 (IBM Corp., Armonk, NY, USA) and R version 4.5.2 (R Foundation for Statistical Computing, Vienna, Austria). A two-sided *p*-value < 0.05 was considered statistically significant unless stated otherwise.

## 3. Results

### 3.1. Patient Characteristics

The analysis was conducted on 49 patients, who collectively underwent 149 study visits during a median follow-up time of 6.55 years. The baseline characteristics are summarised in Table 1, stratified by sex. The median age of the study population was 47 years (IQR 36–57), and 61% of the subjects were female. Left ventricular hypertrophy was observed in 51% of patients. 30.6% presented with intermediate involvement, and 36.7% with advanced involvement. Specific FD therapy was administered in 65.4% of individuals, including enzyme replacement therapy in 36.8% and chaperone therapy in 28.6%.

### 3.2. Association of Adipokines with NT-proBNP and Cardiac Involvement

**Figure 1:**
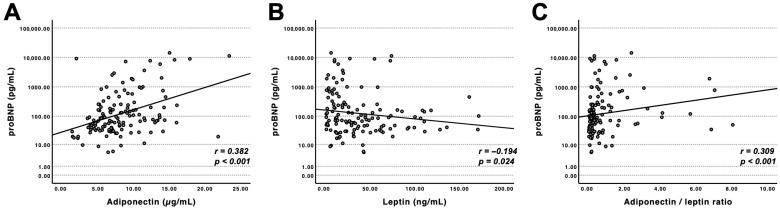
(**A**) Adiponectin was positively correlated with NT-proBNP (r = 0.382, *p* < 0.001). (**B**) Leptin showed an inverse correlation (r = −0.194, *p* = 0.024). (**C**) The adiponectin/leptin ratio was positively associated with NT-proBNP (r = 0.309, *p* < 0.001). Spearman’s rank correlation coefficients are shown. NT-proBNP is displayed on a logarithmic scale to enhance visual clarity (n = 135).

Adiponectin demonstrated a moderate positive correlation with NT-proBNP (r = 0.38, *p* < 0.001), whereas leptin was inversely associated with NT-proBNP (r = −0.19, *p* = 0.02). The adiponectin/leptin ratio was likewise positively correlated with NT-proBNP (r = 0.31, *p* < 0.001), albeit with a lower correlation coefficient compared to adiponectin alone (Figure 1). Furthermore, adiponectin, leptin, and the adiponectin/leptin ratio correlated with high-sensitive troponin T (r = 0.2, *p* = 0.01). Interestingly, no correlations between Adiponectin and left ventricular mass index, interventricular septal thickness, ejection fraction, septal T1 values, estimated glomerular filtration rate, the albumin-to-creatinine ratio, or lyso-Gb3 could be found. Nevertheless, correlations of left ventricular mass index, interventricular septal thickness, and estimated glomerular filtration rate with leptin and the adiponectin/leptin ratio were significant (*p*-values and correlation coefficients are displayed in Supplemental Table S1).

To evaluate whether adiponectin was independently associated with NT-proBNP, a multivariable linear regression model was used, with log-transformed NT-proBNP as the dependent variable. After adjustment for age, sex, BMI, and eGFR, adiponectin remained independently associated with NT-proBNP (β = 0.224, 95% CI 0.162–0.287, *p* < 0.001). Age, sex, BMI, and eGFR were also significant contributors to the model (Table 2).

**Figure 2:**
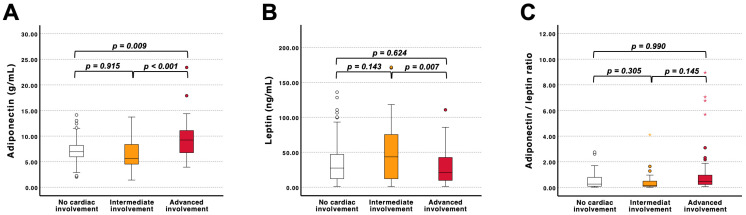
(**A**) Adiponectin, (**B**) leptin, and (**C**) the adiponectin/leptin ratio stratified by no cardiac involvement, intermediate involvement, and advanced involvement. Intermediate cardiac involvement was defined as T1 reduction and/or left ventricular hypertrophy without LGE. Advanced cardiac involvement was defined by the presence of late gadolinium enhancement. *p*-values derived from one-way ANOVA with Bonferroni correction are indicated in the figure. Outliers were marked as stars, whereas extreme values were indicated by circles.

When stratified by degree of cardiac alterations, adiponectin levels differed significantly across groups, with the highest concentrations observed in patients with advanced cardiac involvement (Figure 2A). Leptin levels did not increase with advancing cardiac involvement. Instead, a significant difference was observed between intermediate and advanced stages, with lower levels in advanced disease (Figure 2B). Similarly, the adiponectin/leptin ratio did not differ significantly across groups (Figure 2C). Collectively, these findings indicate that the observed association with NT-proBNP is primarily driven by adiponectin, which may reflect its link to structural cardiac remodeling in FD. When comparing conventional lipid parameters and cardiac imaging, the only significant correlations were found between total and LDL-cholesterol and left ventricular ejection fraction (*p* < 0.01, r = 0.3 and 0.29, respectively).

### 3.3. Sex-Specific Differences and Therapy Status

**Figure 3:**
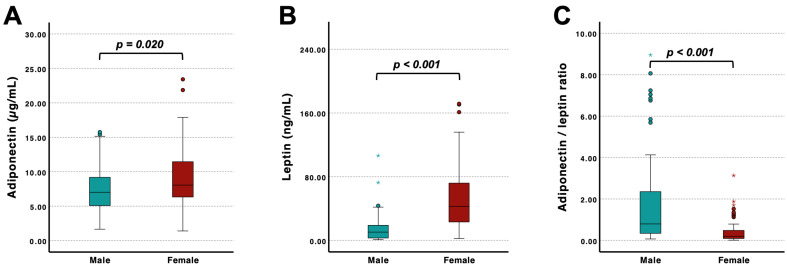
Sex-specific differences in adiponectin, leptin, and the adiponectin/leptin ratio. (**A**) Adiponectin levels were higher in female patients than in male patients (*p* = 0.020). (**B**) Leptin concentrations were significantly elevated in females (*p* < 0.001). (**C**) The adiponectin/leptin ratio differed significantly between sexes (*p* < 0.001). *p*-values derived from group comparisons are indicated in the figure. Outliers were marked as stars, whereas extreme values were indicated by circles.

Sex-specific analyses revealed significantly higher adiponectin levels in female compared to male patients (Figure 3A). Moreover, leptin concentrations were markedly elevated in females (Figure 3B), resulting in a significantly lower adiponectin/leptin ratio (Figure 3C). When stratified by therapy status, these sex-specific differences remained largely preserved (Supplemental Figure S1). In patients without therapy, females exhibited higher adiponectin and leptin levels and a lower adiponectin/leptin ratio than male patients (Supplemental Figure S1A–C). In patients receiving therapy, leptin concentrations and the adiponectin/leptin ratio continued to differ significantly between sexes, whereas adiponectin levels did not differ significantly (Supplemental Figure S1D–F).

Analysis of lipid parameters by therapy status did not reveal significant differences in triglycerides, total cholesterol, HDL cholesterol, or LDL cholesterol, either in the overall cohort or after stratification by sex (Supplemental Figure S4A–D male, Supplemental Figure S4E–H female). When stratified by therapy status, the positive association between adiponectin and NT-proBNP was not significant in untreated patients but was highly significant in patients receiving therapy, with a higher correlation coefficient than in the overall cohort (Supplemental Figure S2A,D). Thus, the association observed in the total cohort appears to be primarily driven by treated patients. The inverse association between leptin and NT-proBNP and the positive association of the adiponectin/leptin ratio were likewise more pronounced in patients receiving therapy (Supplemental Figure S2B,C,E,F).

### 3.4. Longitudinal Adipokine and Lipid Profiles According to Therapy Status and Sex

**Figure 4:**
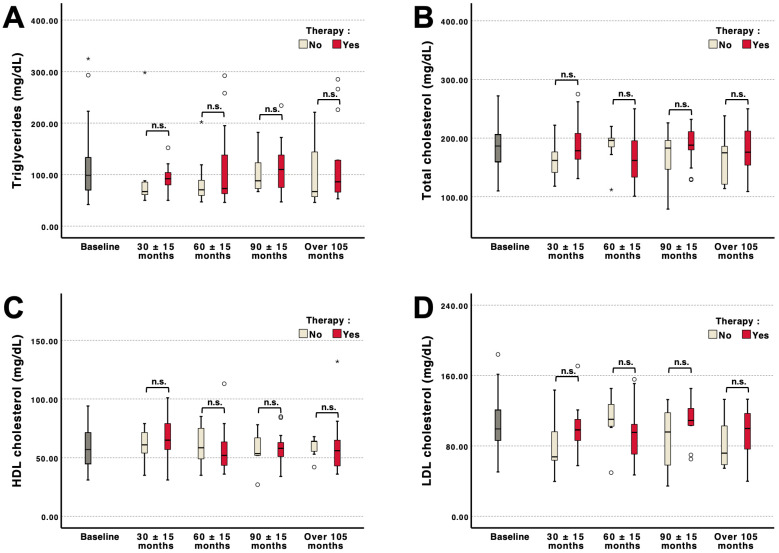
Longitudinal lipid profiles stratified by therapy status. Triglycerides (**A**), total cholesterol (**B**), HDL cholesterol (**C**), and LDL cholesterol (**D**) were assessed at baseline and clustered follow-up visits (30 ± 15, 60 ± 15, 90 ± 15, and >105 months) in patients with and without specific therapy. Baseline included n = 49 observations. At 30 ± 15 months, 22 (8 without vs. 14 with therapy), at 60 ± 15 months n = 33 (13 vs. 20), at 90 ± 15 months n = 20 (7 vs. 13), and at >105 months, n = 25 (7 vs. 18), resulting in 149 total longitudinal observations No significant differences were observed. Outliers were marked as stars, whereas extreme values were indicated by circles.

Longitudinal analysis of adipokines and lipid parameters did not reveal significant differences between patients with and without specific therapy at baseline or during follow-up (Figure 4A–D and Figure S3). Triglycerides, total cholesterol, HDL cholesterol, and LDL cholesterol, as well as adiponectin and leptin, remained comparable between therapy groups across all clustered time points (30 ± 15, 60 ± 15, 90 ± 15, and >105 months), without tangible changes over time.

**Figure 5:**
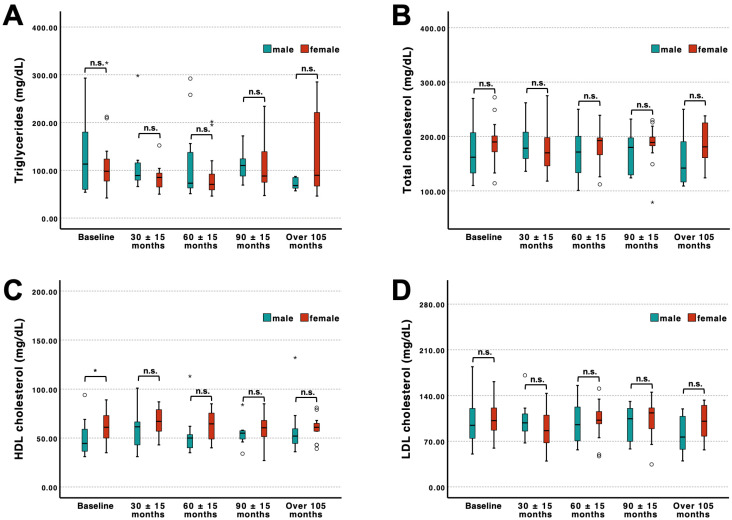
Longitudinal lipid profiles stratified by sex. Triglycerides (**A**), total cholesterol (**B**), HDL cholesterol (**C**), and LDL cholesterol (**D**) were assessed at baseline and clustered follow-up visits (30 ± 15, 60 ± 15, 90 ± 15, and >105 months) in male and female patients. At baseline, n = 19 male and n = 30 female observations were included. At 30 ± 15 months, n = 9 male and n = 13 female, at 60 ± 15 months, n = 13 male and n = 20 female, at 90 ± 15 months, n = 7 male and n = 13 female, and at >105 months, n = 11 male and n = 14 female, resulting in 149 total longitudinal observations. *p*-values for between-sex comparisons at each time point are indicated in the figure. * denotes *p* < 0.05 and “n.s.” denotes non-significant differences. Outliers were marked as stars, whereas extreme values were indicated by circles.

Sex-stratified longitudinal analyses likewise demonstrated largely comparable lipid profiles between male and female patients (Figure 5A–D). Triglycerides, total cholesterol, and LDL cholesterol did not differ significantly between sexes at any time point. Only HDL cholesterol levels were significantly lower in male compared to female patients at baseline (*p* < 0.05), whereas no further significant sex-specific differences were observed during follow-up. Overall, lipid parameters remained stable over time and were not noticeably influenced by therapy status or sex.

## 4. Discussion

Accumulating evidence indicates that adipokines play a significant role in the pathophysiology and prognosis of different cardiomyopathies and heart failure through their regulatory effects on inflammation, metabolism, and myocardial remodeling [17,18,19,20,22]. Although adiponectin is generally considered cardioprotective due to its anti-inflammatory and anti-fibrotic properties, an opposite effect appears to occur in patients with heart failure. Paradoxically, elevated adiponectin levels were found to be closely associated with increased disease severity and mortality [18,22,23,34]. Despite previous reports in FD, we found that higher levels of circulating adiponectin were associated with cardiac involvement in this patient population [24]. Adiponectin correlated positively with NT-proBNP and remained independently associated with log-transformed NT-proBNP values after adjustment for age, sex, BMI, and renal function. In FD, NT-proBNP has previously been suggested to reflect the extent of myocardial hypertrophy and remodeling rather than classical systolic heart failure [27]. Our findings are consistent with this concept and indicate that adiponectin levels might be associated with the degree of myocardial alterations.

Notably, stratified analyses indicated that these observations were primarily attributable to patients receiving FD-specific therapies. While no significant association was observed in untreated individuals, adiponectin showed a strong, significant correlation with NT-proBNP in patients receiving treatment, exceeding that observed in the overall cohort. However, the depicted interrelationship should be interpreted with caution. In clinical practice, disease-specific therapy for FD is typically initiated in patients with more advanced organ involvement, particularly when cardiac manifestations are present. Thus, the observed association may partly reflect confounding by indication rather than a direct treatment-related effect [35,36]. Accordingly, imaging-based stratification further supports this interpretation. Adiponectin was highest in advanced disease presentations, as defined by the presence of LGE on CMR [37,38]. Patients without and with intermediate cardiac involvement exhibited significantly lower concentrations. Enzyme replacement and chaperone therapy may alter the inflammatory process caused by FD. By reducing the glycosphingolipid load, which serves as a substrate for chronic inflammation, both therapies could reduce chronic inflammation [39,40,41,42,43]. However, a consistent suppression of inflammatory activity may not be achieved by currently available therapies. In selected patients, particularly males with nonsense mutations who develop anti-drug antibodies, there is evidence that immunological activation, including complement activation and cytokine release, may even be enhanced during ERT [15,40,44]. This alteration of the inflammatory state could potentially also influence the adipokine secretion. Further studies are needed to differentiate the effects of residual or enhanced inflammation, whether due to or despite FD-specific therapy.

In contrast, leptin did not demonstrate a comparable pattern across imaging-defined stages. Moreover, the adiponectin/leptin ratio exhibited a weaker correlation coefficient than adiponectin alone, suggesting that the observed significance is primarily driven by adiponectin. Taken together, these data position adiponectin as a biomarker potentially linked to cardiac involvement in FD. Its independent association with NT-proBNP and its elevation in patients with LGE-positive cardiomyopathy support its relationship with structural cardiac manifestations. However, whether this represents a compensatory response or a distinct pathophysiological mechanism remains unclear and requires further mechanistic studies to be clarified.

T1-mapping by CMR is an important tool for distinguishing between different hypertrophic cardiomyopathy phenocopies, including FD [45]. Yet no correlations between adiponectin or leptin and septal T1 values, reflecting glycosphingolipid accumulation, were observed. A possible explanation for the missing correlation despite significant group differences across the stages of Fabry cardiomyopathy is T1 pseudo-normalization during the disease process, in which initially decreased T1 values increase as fibrosis forms [46,47]. While T1- and T2-mapping are now widely available for assessing cardiomyopathy in Fabry disease, the range of complementary imaging techniques and image-analysis tools continues to expand. Future disease classification concepts should therefore incorporate a comprehensive evaluation of conventional CMR-derived parameters together with emerging markers, including novel functional markers, epicardial adipose tissue, fibroblast activation imaging, and abnormalities of cardiac sympathetic innervation [7,37,48,49,50,51].

In addition to cardiac associations, we observed pronounced sex-specific differences in adipokine profiles. Female patients exhibited significantly higher leptin levels and a lower adiponectin/leptin ratio compared with males, whereas adiponectin concentrations were significantly higher in females. These findings are in line with known physiological sex differences in adipokine regulation and body fat distribution [52,53]. Importantly, this pattern was consistent across therapy status, indicating that the observed effects persisted despite treatment exposure. However, an interesting finding emerged within the therapy subgroup. While sex differences in leptin and the adiponectin/leptin ratio remained unchanged, the difference in adiponectin levels between males and females was no longer statistically significant. Although exploratory, this finding may suggest that disease levels converge across sexes at more advanced stages of FD. Given the X-linked inheritance of FD, with hemizygous males typically exhibiting more severe and earlier organ involvement, such convergence could reflect a more pronounced or accelerated pathophysiological state in affected male patients [1]. However, whether this represents differential disease burden, therapy-related modulation, or altered compensatory mechanisms lies beyond the scope of the present study. Moreover, X-inactivation patterns, leading to individual women being as severely affected as men, should be taken into account [54]. When examining lipid parameters, no significant differences were observed between treated and untreated patients in triglycerides, total cholesterol, HDL cholesterol, or LDL cholesterol. This lack of association persisted after stratification by sex. Summarizing, these findings indicate that adipokine alterations exhibit a clear sex-dependent pattern, whereas standard lipid parameters appear largely unaffected by therapy status or sex. Yet the modulation of adiponectin in treated males may warrant attention, particularly given the known sex-related differences in FD severity.

In contrast to the associations observed for adipokines, lipid parameters remained largely unchanged over long-term follow-up. Triglycerides, total cholesterol, HDL cholesterol, and LDL cholesterol did not demonstrate significant longitudinal variation, irrespective of therapy status. Thus, within the observed time frame, lipid profiles appear stable and not substantially modified by specific therapy. This is in line with a previous study by Stepien et al. [55]. The discussed observation is particularly relevant given the increased cardiovascular event burden in FD, including cerebrovascular events [2]. Sex-stratified analyses yielded similar results. Aside from lower baseline HDL cholesterol in male patients, no consistent sex-related differences emerged over time. Lipid trajectories remained comparable between males and females, indicating that sex does not meaningfully influence longitudinal lipid changes in our cohort. Lower levels of total and LDL cholesterol were associated with reduced left ventricular ejection fraction, but no links were found with other imaging markers, including those for left ventricular hypertrophy. Taken together, our as well as previously published data do not indicate that changes in conventional lipid parameters substantially contribute to the increased cardiovascular event burden in FD [55,56,57]. Instead, these events may result from disease-specific mechanisms, including intracellular accumulation of glycosphingolipids, structural cardiac involvement, and endothelial dysfunction [58,59].

However, several limitations should be acknowledged. Although our cohort of 49 patients represents a relatively large single-center Fabry population, subgroup analyses, particularly with respect to therapy status and cardiac involvement stage, remain limited and should be interpreted with caution. FD-specific therapy was changed or initiated in individual patients during the observational period due to evolving treatment options and clinical circumstances. Longitudinal analyses were performed using clustered follow-up intervals rather than mixed-effects models. While this approach reflects the real-world follow-up structure of the cohort, more advanced repeated-measures modeling could provide additional granularity. NT-proBNP was used as a marker of cardiac involvement. Despite adjustment for relevant confounders, it may not be disease-specific and may be influenced by renal function and comorbidities. In addition, the lack of association with structural echocardiographic parameters may indicate that adiponectin reflects biochemical markers of cardiac stress and alterations more readily captured by CMR [49]. Finally, lipid analyses were restricted to conventional parameters, and more detailed lipoprotein profiling or vascular function assessment was not performed. Lyso-Gb3 measurements were not obtained in the initial phase of the KarMA registry and laboratory schemes changed during the observational period, thus leading to missing data and consequently limiting statistical power.

## 5. Conclusions

In this longitudinal cohort study of patients with FD, adiponectin was independently associated with NT-proBNP and increased with advancing imaging-defined cardiac involvement, particularly in treated patients. These findings suggest that adiponectin may be associated with structural cardiac manifestations in FD. In contrast, leptin showed weaker and less consistent associations. Conventional lipid parameters remained stable over long-term follow-up and were not meaningfully influenced by therapy status or sex. Our data, therefore, do not support a significant contribution of progressive dyslipidemia to the cardiovascular phenotype observed in FD. Overall, alterations in adiponectin and, to a lesser degree, in leptin appear to reflect cardiac involvement, whereas traditional lipid profiles remained largely unchanged over time.

## Figures and Tables

**Table 1:** Patient characteristics at the most recent study visit.

Variable	Overall *N* = 49	Male *N* = 19	Female *N* = 30	*p*-Value
**Age (years)**	47 (36–57)	47 (38–66)	47 (29–57)	0.167
**Follow-up time (years)**	6.55 (±44.62)	8.18 (±4.89)	5.56 (±4.24)	0.082
**Specific therapy**				
No therapy	17 (34.6%)	3 (15.8%)	14 (46.7%)	
Agalsidase alfa	16 (32.7%)	8 (42.1%)	8 (26.7%)	
Agalsidase beta	2 (4.1%)	1 (5.3%)	1 (3.3%)	
Migalastat	14 (28.6%)	7 (36.8%)	7 (23.3%)	
**Type of cardiac involvement**				
None	16 (32.7%)	4 (21.1%)	12 (40%)	
Intermediate (LVH or low T1)	15 (30.6%)	7 (36.8%)	8 (26.7%)	
Advanced (LGE on CMR)	18 (36.7%)	8 (42.1%)	10 (33.3%)	
**Laboratory markers**				
Adiponectin (µg/mL)	7.36 (5.31–9.9)	5.78 (4.35–8.99)	8.21 (5.65–10.26)	**0.020**
Leptin (ng/mL)	39.1 (11.4–56.4)	11.4 (5.34–15.1)	48.6 (32.9–70.6)	**<0.001**
Adiponectin/Leptin ratio	0.25 (0.11–0.77)	0.77 (0.34–2.06)	0.17 (0.09–0.32)	**<0.001**
Total cholesterol (mg/dL)	188 (144–206)	156 (137–200)	189 (170–212)	0.233
LDL-cholesterol (mg/dL)	107 (75.4–120)	89.2 (71.6–120)	107.8 (86–120)	0.558
HDL-cholesterol (mg/dL)	58 (43–67.5)	47.5 (36.3–62.8)	62 (53–75)	**0.016**
Lipoprotein(a) (nmol/L)	27 (10–131)	30 (11.3–120)	27 (10–136)	0.678
Triglyceride (mg/dL)	86 (67–120)	85 (70.8–121)	88 (67–111)	0.835
High-sensitive Troponin T (ng/L)	13 (5–30)	33 (11–61)	6.5 (4–18.8)	**<0.001**
NT-proBNP (pg/mL)	101 (39.5–275)	327 (72–1175)	65.5 (37.1–158.9)	**0.022**
Creatinine (mg/dL)	0.8 (0.72–0.96)	0.96 (0.86–1.49)	0.74 (0.66–0.8)	**0.001**
eGFR (mL/min/1.73 m^2^)	93.78(72.8–112)	86.7 (57.9–99)	98.3 (80–114)	0.095
Albumin-to-creatinine ratio (mg/g)	23 (9–62)	27.5 (11–340)	16 (7.5–58)	0.406
Lyso-Gb3 (ng/mL)	4.7 (2–32.4)	34.75 (33.6–35.9)	2 (2–3.4)	**0.025**
**Cardiac imaging- CMR**				
Left ventricular mass index(g/m^2^)	63.9 (61.4–81.5)	119 (94.6–141)	62.5 (59.6–64.6)	0.018
Left ventricular EF (%)	60 (57–66.9)	59.9 (53.4–62.2)	62 (57.1–67)	0.186
Right ventricular EF (%)	54.6 (52–61.9)	59.3 (54–60.2)	54.1 (52.8–65.2)	0.889
LVH (n)	25 (51%)	11 (57.9%)	14 (46.7%)	0.561
Septal T1 (ms)	987 (±75.6)	949(±114.6)	999(±62)	0.529
**Cardiac imaging- Echocardiography**				
IVS thickness (mm)	11 (10–16)	15 (11–20)	10.5 (10–13)	**<0.001**
**ECG**				
Normal (%)	15 (30.6%)	1 (5.3%)	14 (46.7%)	
Sinus bradycardia (%)	7 (14.3%)	2 (10.5%)	5 (16.7%)	
Short PQ (%)	9 (18.4%)	4 (21.1%)	5 (16.7%)	
Atrial fibrillation/Atrial flutter (%)	1 (2%)	0 (0%)	1 (3.3%)	
Bundle branch blocks (%)	7 (14.3%)	4 (21.1%)	3 (10%)	
AV-Block I° (%)	1 (2%)	1 (5.3%)	0 (0%)	
Higher degree AV-Block (II° and III°) (%)	0 (0%)	0 (0%)	0 (0%	
Left ventricular hypertrophy * (%)	18 (36.7%)	10 (52.6%)	8 (26.7%)	
Repolarization abnormalities (%)	19 (38.8%)	8 (42.1%)	11 (36.7%)	
Pacemaker (%)	4 (8.2%)	4 (21.1%)	0 (0%)	

LVH, left ventricular hypertrophy; LGE, late gadolinium enhancement; CMR, cardiac magnetic resonance; EF, ejection fraction; IVS, interventricular septum; ECG, electrocardiogram; A *p*-value of <0.05 was considered statistically significant (bold). Data is displayed as count + percent, mean ± standard deviation or median + interquartile range. * Left ventricular hypertrophy was defined as an Sokolow–Lyon index > 3.5 mV.

**Table 2:** Multivariable linear regression analysis for log-transformed NT-proBNP.

Variable	β (SE)	95% CI	*p*-Value
Adiponectin (µg/mL)	0.224 (0.031)	0.162–0.287	**<0.001**
Age (years)	0.023 (0.009)	0.005–0.041	**0.013**
Sex	−1.261 (0.245)	−1.747–−0.774	**<0.001**
BMI (kg/m^2^)	0.120 (0.027)	0.068–0.173	**<0.001**
eGFR (mL/min/1.73 m^2^)	−0.015 (0.004)	−0.023–−0.006	**0.001**

Multivariable linear regression analysis evaluating the association between adiponectin and log-transformed NT-proBNP. β coefficients with standard errors (SE), 95% confidence intervals (CI), and corresponding *p*-values are shown. The model was adjusted for age, sex, BMI, and estimated glomerular filtration rate (eGFR). A *p*-value of <0.05 was considered statistically significant (bold). Model performance: R^2^ = 0.681; adjusted R^2^ = 0.663; F = 37.97; *p* < 0.001.

## Data Availability

The raw data supporting the conclusions of this article will be made available by the authors upon reasonable request.

## Supplementary Materials

The following supporting information can be downloaded at: https://www.mdpi.com/article/10.3390/jcm15062390/s1. Figure S1: Sex-specific differences in adiponectin, leptin, and the adiponectin/leptin ratio stratified by therapy status. Figure S2: Correlation of adiponectin, leptin, and the adiponectin/leptin ratio with NT-proBNP stratified by therapy status. Figure S3. Longitudinal adipokine profiles stratified by sex and therapy status. Supplemental Figure S4. Lipid parameters according to therapy status stratified by sex. Supplemental Table S1. Correlations of adiponectin and leptin with Age and established Fabry disease biomarkers.
