# Supplementary material for: Longitudinal Adipokine and Lipid Profiles in Fabry Disease
Source: J Clin Med. 2026 Mar 20;15(6):2390. doi: 10.3390/jcm15062390 (PMC13028138; doi:10.3390/jcm15062390)
Supplement: Supplementary file 1 [file jcm-15-02390-s001.zip › jcm-4211292-supplementary.pdf]

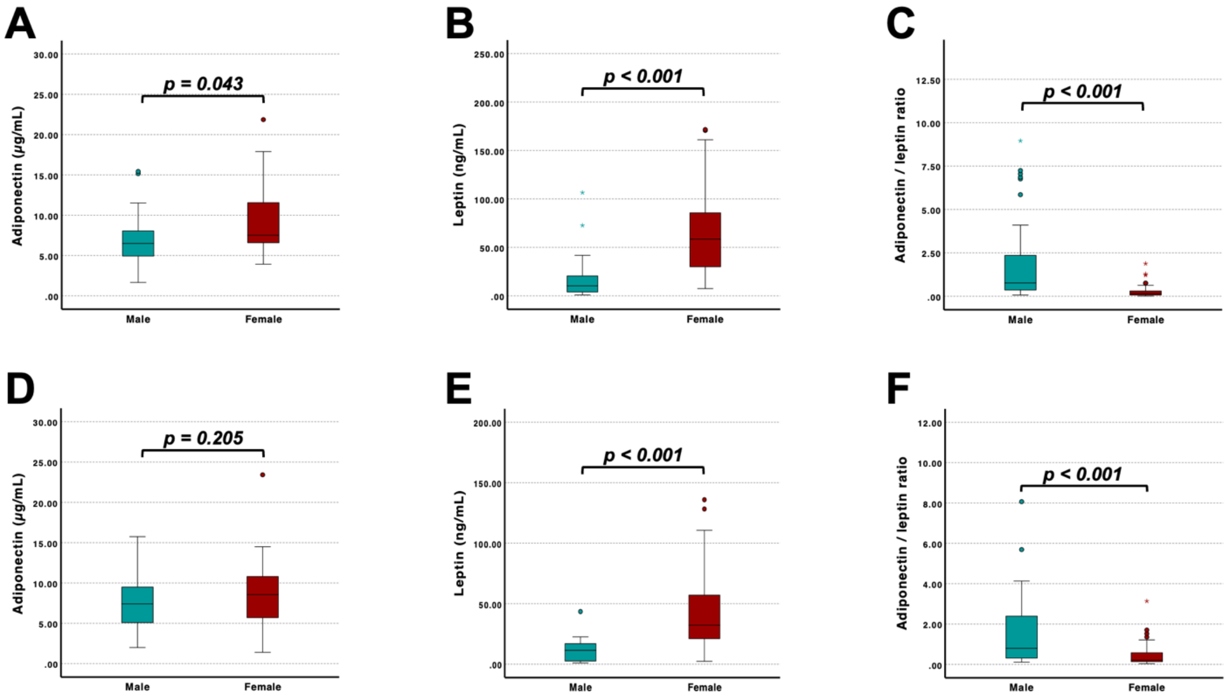

Supplemental Figure S1. Sex-specific differences in adiponectin, leptin, and the adiponectin/leptin ratio stratified by therapy status. (A–C) Patients without therapy, (D–F) patients receiving specific therapy. (A, D) Adiponectin, (B, E) leptin, and (C, F) the adiponectin/leptin ratio stratified by sex. P-values derived from group comparisons are indicated in the figure.

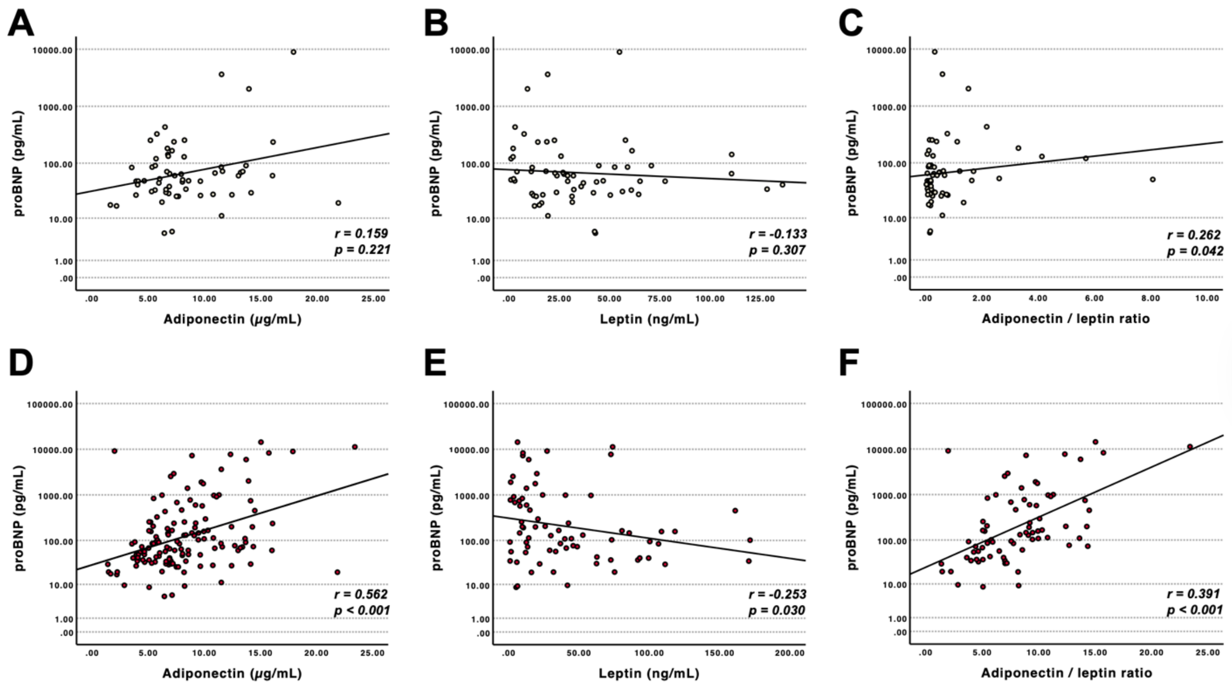

Supplemental Figure S2. Correlation of adiponectin, leptin, and the adiponectin/leptin ratio with NT-proBNP stratified by therapy status. (A–C) Patients without therapy, (D–F) patients receiving specific therapy. (A, D) Adiponectin, (B, E) leptin, and (C, F) the adiponectin/leptin ratio in relation to NT-proBNP. Spearman's rank correlation coefficients are presented. NT-proBNP values are displayed on a logarithmic scale to enhance visual clarity. P-values are indicated in the figure.

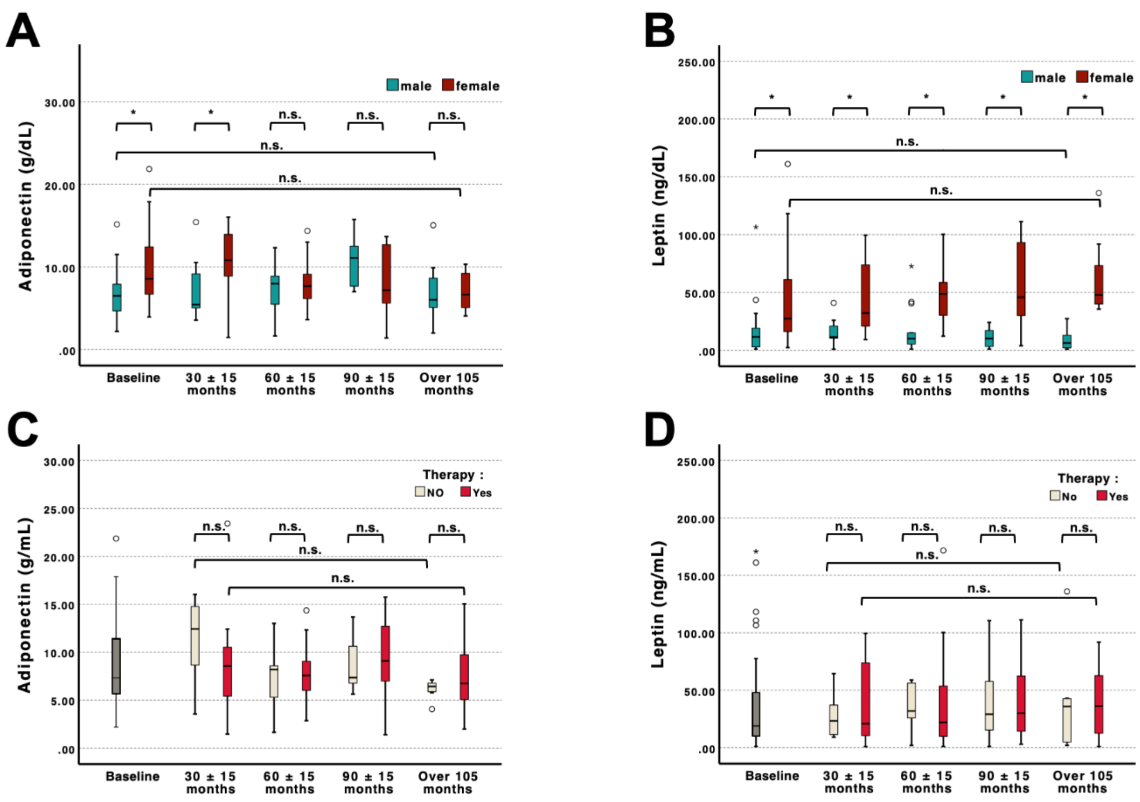

Supplemental Figure S3. Longitudinal adipokine profiles stratified by sex and therapy status. (A) Adiponectin and (B) leptin concentrations assessed at baseline and clustered follow-up visits (30 ± 15, 60 ± 15, 90 ± 15, and >105 months) stratified by sex. (C) Adiponectin and (D) leptin concentrations were assessed at the same time points, stratified by therapy status (no specific therapy vs. specific therapy). Comparisons across time points were performed using one-way ANOVA with Bonferroni correction. * denotes p < 0.05 and "n.s." denotes non-significant differences.

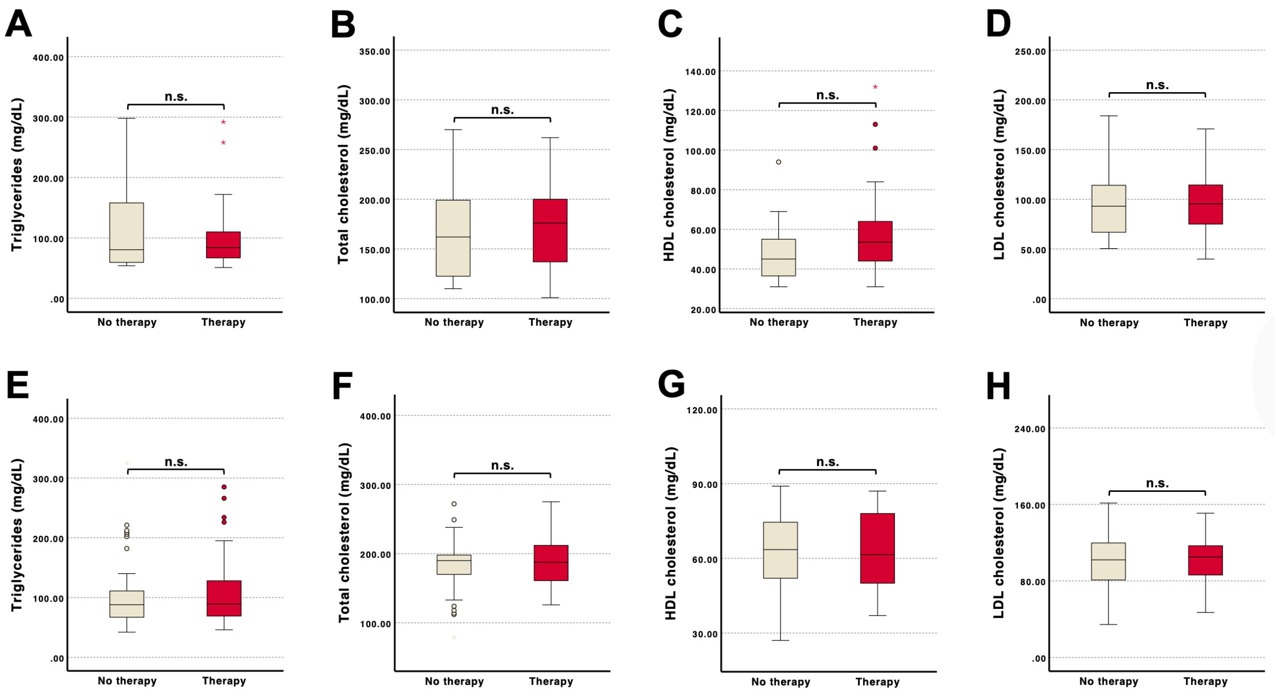

Supplemental Figure S4. Lipid parameters according to therapy status stratified by sex. (A–D) Male patients, (E–H) female patients. Triglycerides (A, E), total cholesterol (B, F), HDL cholesterol (C, G), and LDL cholesterol

(D, H) are shown for patients without therapy and with therapy. No significant differences were observed between treatment groups in either sex (male n=50, female n=83).

Supplemental Table S1. Correlations of adiponectin and leptin with age and established Fabry disease biomarkers

	**Adiponectin**	**Leptin**	**Adiponectin/leptin ratio**
Age	**r=0.28, p<0.001**	r=-0.13, p=0.12	**r=0.2, p=0.01**
NT-proBNP	**r=0.38, p<0.001**	**r=-0.19, p=0.02**	**r=0.31, p<0.001**
High sensitiv troponin T	**r=-0.2, p=0.013**	**r=-0.24, p<0.01**	**r=0.27, p<0.001**
LVMI	r=-0.07, p=0.51	**r=-0.34, p<0.01**	**r=0.28, p<0.014**
Interventricular septal thickness	r=0.18, p=0.051	**r=-0.25, p<0.01**	**r=0.29, p<0.01**
Ejection fraction	r=0.05, p=0.09	r=-0.16, p<0.07	r=0.16, p=0.08
Lyso-Gb3*	r=0.11, p=0.54	r=-0.34, p=0.05	r=0.3, p=0.08
eGFR	r=-0.16, p=0.052	**r=-0.09, p=0.28**	**r=0.03, p=0.74**
Albumin-to-creatinine ratio	r=0.04; p=0.068	r=0.05, p=0.65	r=-0.01, p=0.89

r, Spearman's rank correlation coefficient; LVMI, left ventricular mass index; eGFR, estimated glomerular filtration rate (calculated using the CKD-EPI equation)[1]. A p-value of <0.05 was considered statistically significant (bold) *n=33

1. Inker, L.A., et al., *New Creatinine- and Cystatin C-Based Equations to Estimate GFR without Race.* N Engl J Med, 2021. **385**(19): p. 1737–1749.
